# ASSOCIATION OF COGNITIVE LEVEL AND QUALITY OF LIFE INDICATORS POSTSTROKE IN OLDER ADULTS: A CROSS-SECTIONAL STUDY

**DOI:** 10.1093/geroni/igae098.3036

**Published:** 2024-12-31

**Authors:** Azza Alharrasi, Diane Berish

**Affiliations:** The Pennsylvania State University, University Park, Pennsylvania, United States; The Pennsylvania State University, University Park, Pennsylvania, United States

## Abstract

Stroke is one of the leading causes of death and disability worldwide. Post-stroke cognitive impairment (PSCI) is a common complication of stroke; however, mild cognitive impairment is highly prevalent within three months post-stroke and is associated with increased cost of care, hospitalizations, and care dependency. This study examined the association between cognition level (TICS total score) and quality of life indicators (Depression yes=1 no=0, physical limitations yes=1 no=0, socialization yes=1 no=0) among adults aged 65+. Therefore, a cross-sectional study using the 2020 wave of Health and Retirement Study (HRS) was used to conduct logistic regression Analysis using R.4.2.2. Total sample was 1259 adults 65+ who had reported experiencing a stroke, 58% were female and average TICS score of 6.5 (SD=3.5). Covariates of having high blood pressure, diabetes, or heart disease, sex, working outside the home, education level and age were included in all models. For every one-point increase in TICS score the odds of experiencing functional impairment were 21% lower (OR =0.89, p <.0001). The association for TICS scores and odds of feeling depressed (OR=0.97, p=0.27) and odds of socialization (OR 1.02, p=0.68) were not statistically significant. These results indicate that older adults who had lower cognition scores post stroke were more likely to experience physical limitations. More rigorous research studies need to be conducted to evaluate the quality of life of older adults during post-stroke based on their cognition status. This will inform medical management to individualize the rehabilitation and treatment according to the older adults’ needs.

